# Analysis of cultivation conditions and enzymatic efficiency enables high-yield levan production from *Bacillus velezensis* KKSB6

**DOI:** 10.1186/s40643-025-00994-2

**Published:** 2025-12-26

**Authors:** Wirada Kaewyotha, Komsan Kaewmuang, Nuntavun Riddech, Wiyada Mongkolthanaruk

**Affiliations:** Department of Microbiology, Faculty of Science, Khon Kaen University, Khon Kaen, 40002 Thailand

**Keywords:** Biopolymer, Bacterial fermentation, Enzymatic levan production, Exopolysaccharide, Levansucrase

## Abstract

**Abstract:**

Levan, a fructose-based biopolymer with significant industrial potential, was produced using the native strain *Bacillus velezensis* KKSB6. This study aimed to optimize levan production through different cultivation strategies and to characterize the enzyme responsible, levansucrase. Production was compared in batch, fed-batch and continuous systems, revealing a critical point between final yield and productivity. Batch cultivation achieved a high levan yield of 187 g/L (93.50% yield), while a fed-batch cultivation delivered high productivity at 3.46 g/L/h but a low levan yield (55.33%). Continuous cultivation demonstrated the highest levan production of 746 g/L (93.25% yield) and high productivity (7.77 g/L/h). In those systems, production was limited by the inhibitory effect of glucose accumulation. The purified levansucrase, a 42 kDa protein, exhibited optimal activity at 35 °C and pH 7.0. Significantly, in vitro synthesis using the purified enzyme produced primarily high-molecular-weight (HMW) levan, in contrast to the lower-molecular-weight product obtained from whole-cell fermentation. In conclusion, *B. velezensis* KKSB6 is an exceptionally potent strain, with batch cultivation or continuous cultivation producing levan yield and with continuous cultivation favoring productivity. The purified enzyme represents a promising biocatalyst for the targeted synthesis of HMW levan.

**Graphical abstract:**

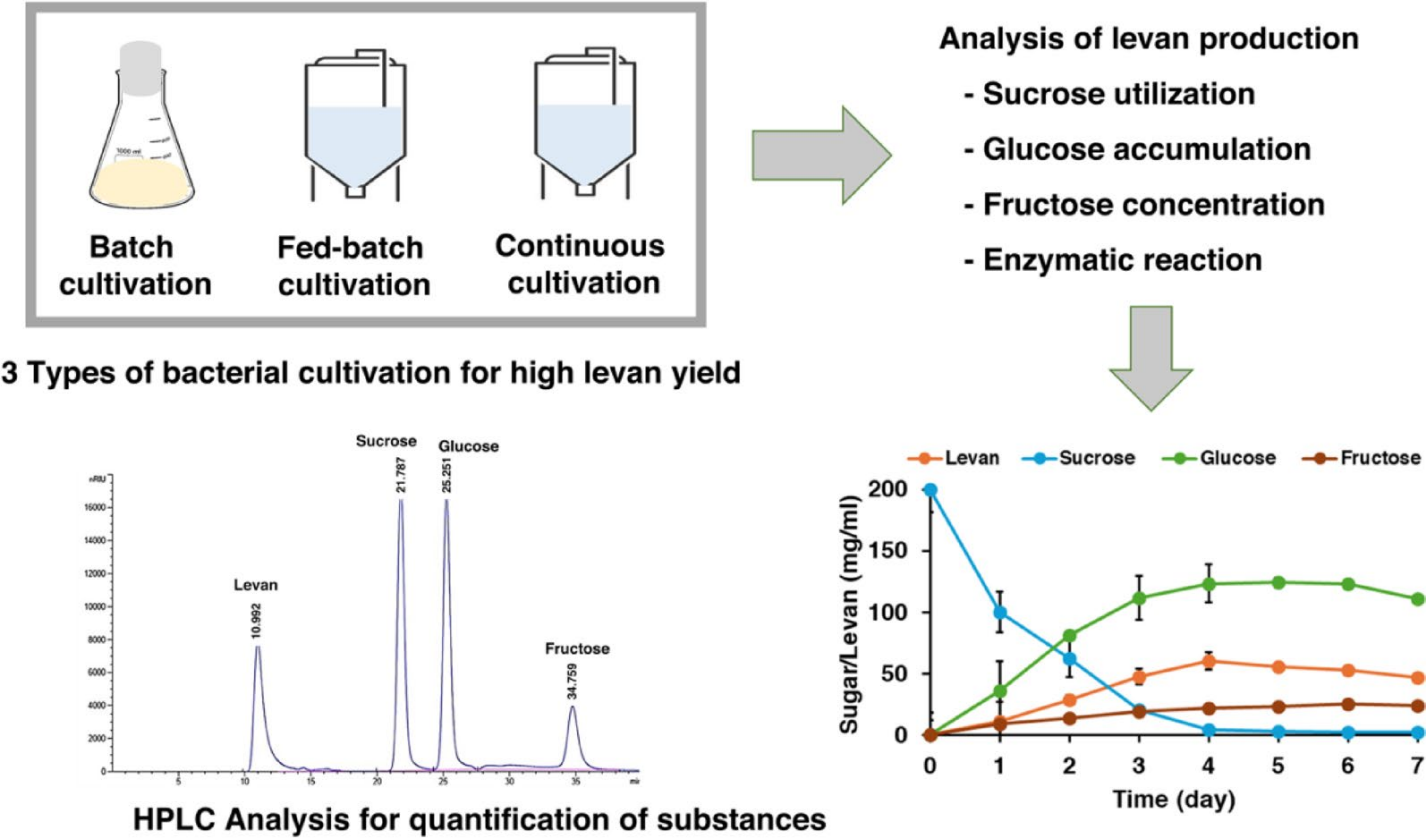

**Supplementary Information:**

The online version contains supplementary material available at 10.1186/s40643-025-00994-2.

## Introduction

Levan is a homopolysaccharide consisting of repeating units of fructose connected by β-2,6-glycosidic linkages, and is a biodegradable, non-toxic and highly biocompatible compound. It is utilized as an emulsifying, stabilizing and thickening agent, in addition to being used a sweetener in the food industry (Bersaneti et al. 2018). Levan can also be combined with other compounds for use in food packaging materials (Gan et al. 2022). Moreover, its high-value properties are utilized in the medical and pharmaceutical industry, for example, in the stimulation of the proliferation of human keratinocytes and fibroblasts, for control of drug release, and in the form of nanoparticles and hydrogels (Kim et al. 2003; Cinan et al. 2021; Demirci et al. 2020). The molecular weight of levan is directly related to its properties. High molecular weight levan exhibits anticancer activity and lowers cholesterol, while low molecular weight levan has the ability to encapsulate various substances and possesses antioxidant properties (Domżał-Kędzia et al. 2023).

Levansucrase (EC 2.4.1.10), also referred to as β-D-fructofuranosyl transferase, is an enzyme that catalyzes the transfer of the fructosyl group from sucrose to create β-2,6-linked oligosaccharides and levan chains. This enzyme belongs to the GH68 family, which is found in various bacterial genera including *Acetobacter*, *Bacillus*, *Clostridium*, *Geobacillus*, *Lactobacillus*, *Leuconostoc*, *Pseudomonas*, *Zymomonas*, Erwinia, *Gluconobacter*, *Gluconacetobacter*, and *Halomonas* (Lekakarn et al. 2024). Levansucrase is encoded by the *sacB* gene which isoften located adjacent to the *yveB* gene (encoding an enzyme with levanase activity) and is secreted via the SecA pathway (Daguer et al. 2004). While sucrose serves as a substrate for levan synthesis, its optimal concentration varies between different bacterial species. Furthermore, glucose accumulating cultivation is a critical problem in levan production; achieving high yields at a low cost is necessary for the commercial application of levan produced via microbial fermentation.

*Bacillus velezensis* KKSB6 was isolated and the optimal conditions for high levansucrase activity were characterized, revealing the optimal cultivation at 200 g/L of sucrose concentration, 37 °C, pH 6 (Wannasutta et al. 2025). This current research aimed to examine sucrose utilization and glucose accumulation during *Bacillus velezensis* KKSB6 growth in batch, fed-batch and continuous cultivations, with the goal of achieving high efficiency, productivity and conversion rates in order to enhance levan production. Additionally, the purified levansucrase was investigated to determine the optimal activity conditions; its potential to produce levan directly from the enzymatic reaction was also evaluated to compare the processes of levan production between using bacterial cultivation or using enzyme reaction in vitro to receive high levan yield. As the cultivation had negative effects of by-product (glucose concentration); using purified enzyme directly maybe produce higher levan yield. Furthermore, the molecular weight of levan might be affected by different processes. This work indicated the suitable process for both high-molecular-weight levan and low-molecular-weight fructo-oligosaccharides (FOS) production.

## Materials and methods

### Bacterial strain and inoculum preparation

*B. velezensis* KKSB6, previously isolated from fermented soybean (Thua nao), was identified as a potential strain for levan production using sucrose as a substrate (Wannasutta et al. 2025). This strain was grown overnight in Luria Bertani (LB) broth until it reached an optical density (OD_600_) of 0.8 to be used as a starter culture. An aliquot of the starter culture (10% v/v) was then inoculated into the main production medium (LB broth supplemented with 200 g/L of sucrose) and incubated at 37 °C with agitation at 150 rpm. At specified time points, culture samples were collected to measure bacterial growth (OD_600_) and were then centrifuged (8000 rpm, 4 °C, 5 min) to separate the supernatant for subsequent analysis of levansucrase activity and levan concentration.

### Batch, fed-batch and continuous cultivation for levan production

For batch cultivation, *B. velezensis* KKSB6 was grown in 150 mL of LB broth supplemented with 200 g/L of sucrose in a 600 mL flask, and incubated at 37 °C with agitation at 150 rpm. Samples were collected at 12, 24, 36, 48, 60, 72, 96 and 120 h for the determination of growth, enzyme activity and levan concentration. For fed-batch cultivation, two strategies were employed. In the first strategy, fresh medium (50 mL of LB with 200 g/L of sucrose) was added to the culture once at 24, 48–72 h. In the second strategy, the medium was added 3 times (24, 48 and 72 h). Lastly, continuous cultivation was performed by adding fresh medium (50 mL of LB with 400 g/L of sucrose) whilst simultaneously removing 50 mL of the cultivation at 24, 48 and 72 h. The samples in each cultivation were collected at 24, 48, 72, 96, 120, 144 and 168 h for determination of growth, enzyme activity, sugar content and levan concentration using HPLC analysis.

### Levansucrase activity assay

Levansucrase activity was determined by measuring the release of reducing sugars from sucrose. 0.5 mL of cell free culture supernatant was mixed with 0.5 mL of 1% (w/v) sucrose solution in 50 mM acetate buffer (pH 6.0). The reaction was incubated at 30 °C for 10 min, followed by the addition of 0.5 mL of DNS reagent to terminate the reaction (Xu et al. 2021). The mixture was then boiled for 10 min and subsequently cooled in an ice bath. The amount of reducing sugar produced was determined using 3,5-dinitrosalicylic acid (DNS) method (Miller 1959) by measuring absorbance at 540 nm with a spectrophotometer. The final volume was adjusted to 5 mL with distilled water before measurement. Glucose was used as a standard for the calibration curve. The negative control was conducted using cell-free culture supernatant without the substrate, and the resulting value was subtracted from the corresponding sample. One unit of levansucrase activity was defined as the amount of enzyme required to released 1 µmol of reducing sugar per min under assay conditions. The highest enzyme activity observed was defined as 100% to calculate relative activity.

### Levan precipitation and HPLC analysis

The supernatant was precipitated by adding 3 volumes of cold 95% ethanol, mixed well by inverting the tube; the mixture was then centrifuged at 8000 rpm, 4 °C for 10 min. The levan pellet was air-dried and stored at 4 °C. For analysis, the levan was dissolved in distilled water and filtered through a 0.45 μm syringe filter. The sample was analyzed using High-Performance Liquid Chromatography (HPLC) equipped with a Gel Permeation Chromatography (GPC) column (Agilent Technologies). The separation was performed on a Hi-Plex Pb column (300 × 7.7 mm) at 80 °C with a flow rate of 0.40 mL/min and a system pressure of 20–23 bar. The mobile phase used distilled water and detection was performed with a Refractive Index Detector (RID). The standards, levan from *Erwinia herbicola* (Sigma-Aldrich), sucrose, fructose and glucose, were analyzed under the same conditions as the samples.

### Levansucrase purification

Crude enzyme was concentrated using Amicon ultra-centrifugal filter (30 kDa cutoff, Merck Millipore) by centrifuging at 5000 rpm, 4 °C, 20 min. After that, the concentrated enzyme was loaded onto a DEAE Sepharose column (HiPrep DEAE FF16/10, GE Healthcare) pre-equilibrated with 50 mM Phosphate buffer (pH 7). The bound proteins were eluted with a linear gradient of NaCl from 0 to 1 M in the same buffer. Fractions were collected at a flow rate of 2 mL/min and protein elution was monitored by measuring absorbance at 280 nm. Levansucrase activity was determined in all collected fractions. The protein content of the purified enzyme fractions was determined using Bradford reagent (Bio-Rad), and purity was assessed by SDS-PAGE. Briefly, the protein sample was mixed with a loading dye and boiled for 5 min before loading onto a 10% separating acrylamide gel. The gel was placed in a running buffer (25 mM Tris-HCl, 200 mM glycine, 0.1% SDS) at 135 voltage for 2 h using OmniPAGE Electrophoresis Systems (Cleaver Scientific Ltd). The gel was stained with Brilliant Blue strain and de-stained with a destaining solution (10% acetic acid and 10% methanol). The protein sample was compared with a standard protein ladder (PiNK Plus Prestained Protein Ladder, GeneDireX, Taiwan).

### Enzyme characterization

The optimal temperature for the purified enzyme was determined by incubating it with 1% (w/v) sucrose at 25, 30, 35 40 and 45 °C for 10 min before performing the DNS assay. For temperature stability, the purified enzyme was incubated at 4, 25 and 35 °C for 30 min, 1, 3 and 6 h. After incubation, residual activity was measured by mixing the enzyme with sucrose at 35 °C for 10 min and performing the DNS assay.

The optimal pH was assessed over a range of pH 5, 6, 7 and 8; the enzyme was mixed with sucrose in buffers of different pH values and incubated at 35 °C for 10 min. For pH stability, the enzyme was pre-incubated in buffers of varying pH for 30 min, 1, 2, 3 and 6 h. Residual activity was then measured by adding sucrose and incubating at 35 °C for 10 min. The amount of reducing sugar was determined by DNS method to calculate levansucrase activity.

The effect of substrate concentration was evaluated using sucrose concentrations of 1, 5, 10, 15 and 20 g/L. The enzyme was mixed with various concentrations of sucrose and incubated at 35 °C for 10 min before the DNS assay. Finally, levan production by the purified enzyme was determined by weight and by HPLC. The reaction mixture contained purified enzyme (1.5 U/mL) and the optimum sucrose concentration (20 g/L) at 35 °C, pH 7.

### Statistical analysis

All experiments were performed in triplicate, and the results are expressed as mean ± standard deviation. Comparisons among groups were conducted using analysis of variance (ANOVA) with STATISTIX 10 software, followed by the least significant difference (LSD) test. A *p*-value < 0.05 was considered statistically significant for multiple comparisons.

## Results

### Comparison of levan production by *B. velezensis* KKSB6 in batch, fed-batch and continuous cultivation

**Fig. 1:**
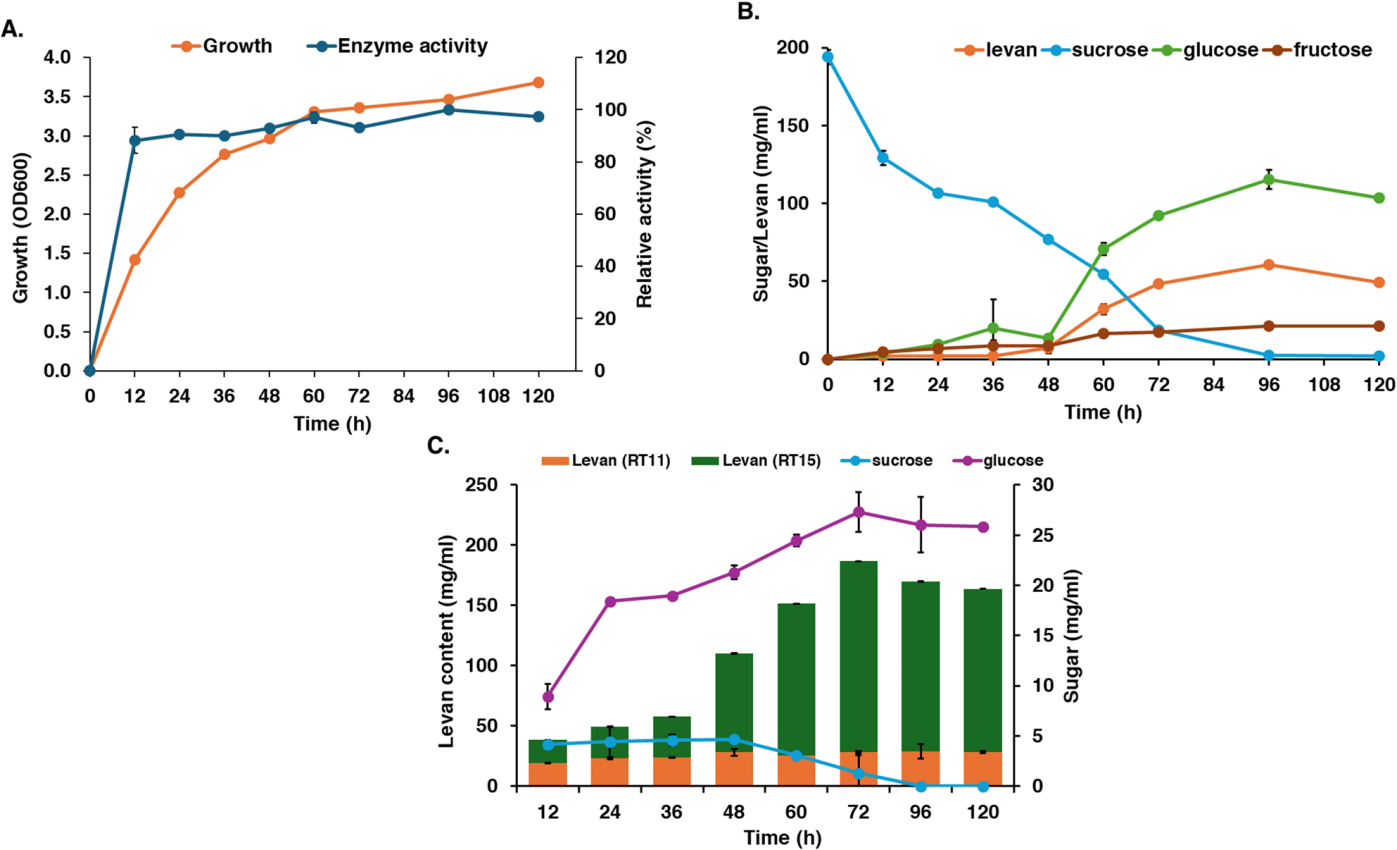
Batch cultivation of *Bacillus velezensis* KKSB6 in LB with 200 mg/ml sucrose at 37 °C. **A** growth and levansucrase activity; **B** sugar concentration and levan in the supernatant of culture; **C** amount of levan pellets detected at retention time of 11 and 15 min (RT11 and RT15, respectively)

In batch cultivation, levansucrase production was observed to be growth-associated with the highest activity recorded at 60 h, which corresponded to the stationary phase of growth (Fig. 1). During cultivation, the sucrose concentration decreased sharply within the first 12 h and was reduced by 50% at 60 h. At this time, glucose began to accumulate in the culture reaching its highest concentration of 115 mg/mL at 96 h. In contrast, fructose concentration remained low (approximately 20 mg/mL) throughout the cultivation. These results indicate that sucrose was hydrolyzed into glucose and fructose; the fructose was rapidly utilized to produce levan which was first detected at 12 h of cultivation. After the sucrose was depleted (at 96 h), the bacteria appeared to utilize the accumulated glucose for growth, which coincided with the termination of levan production. Notably, glucose accumulation to approximately 100 mg/mL appeared to negatively affect levansucrase activity and subsequent levan production. The specific growth rate in batch cultivation was 0.042 h^− 1^; the productivity of levan was 2.60 mg/mL/h, yielding a final concentration of 187 mg/mL of levan after 3 days (Table 1).

**Table 1:** Levan production from batch, fed-batch and continuous cultivation

Cultivation	Sucrose (g/L)	Levan (g/L)	Time (h)	Yield^a^ (%)	Productivity^b^ (g/L/h)
Batch	200	187	72	93.50	2.60
*Fed-batch*					
T1	200 + 100	166	48	55.33	3.46
T2	200 + 100	173	72	57.66	2.40
T3	200 + 100	215	120	71.66	1.79
T4	200 + 100 + 100 + 100	180	72	36.00	2.50
Continuous cultivation	800	746	96	93.25	7.77
Purified enzyme	200	87.67	36	43.84	2.44

**Fig. 2:**
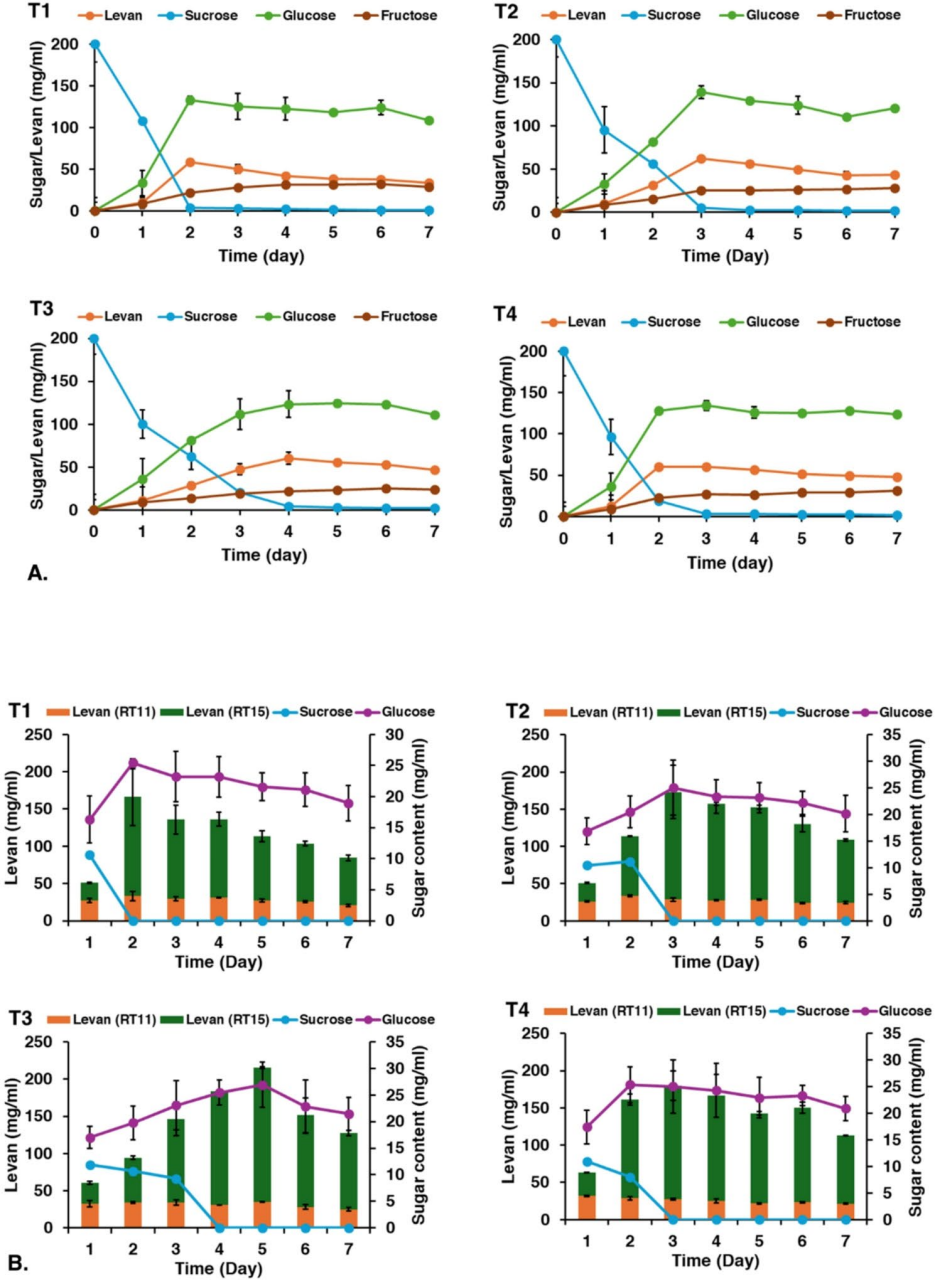
Fed-batch cultivation of *Bacillus velezensis* KKSB6 in LB with 200 mg/ml sucrose at 37 °C that was added the nutrient (LB + 20%sucrose) at 24 h (T1), 48 h (T2), 72 h (T3) and 3 times at 24, 48, 72 h (T4). **A** sugar concentration and levan in the supernatant of culture in each treatment; **B** amount of levan pellets detected at retention time of 11 and 15 min (RT11 and RT15, respectively) in each treatment

For fed-batch cultivation, four different feeding strategies were tested: a single sucrose addition at 24 h (T1), 48 h (T2), or 72 h (T3), and multiple additions every 24 h for three consecutive days (T4). The growth profiles were similar across all treatments, with a slight increase in cell density observed after each sucrose addition. However, the highest overall growth was observed in treatment T1 (sucrose added at 24 h) (Fig. S1). The specific growth rate for all fed-batch treatments were similar, at approximately 0.03 h^− 1^. The patterns of levansucrase activity were also similar among all treatments, suggesting that the timing of feeding had little effect on enzyme production after the initial 48 h. In all cases, the added sucrose was completely consumed within the 24 h after feeding, likely due to the increased cell density requiring more substrate (Fig. 2A). As observed in the batch culture, glucose accumulated to its highest concentration when sucrose was nearly depleted. This again suggests that glucose concentrations exceeding 100 mg/mL had a direct inhibitory effect on levansucrase activity and, consequently, on levan production (Fig. 2B). The highest levan concentrations achieved were 166 mg/mL (T1 at 2 days), 173 mg/mL (T2 at 3 days), 215 mg/mL (T3 at 5 days) and 180 mg/mL (T4 at 3 days). Although treatment T3 produced the highest final levan concentration (yield), treatment T1 exhibited the highest productivity at 3.46 mg/mL/h (Table 1).

**Fig. 3:**
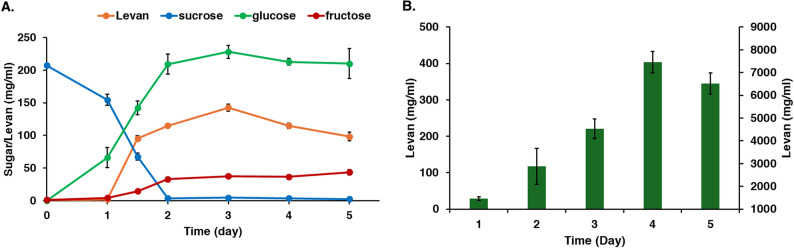
Continuous cultivation of *Bacillus velezensis* KKSB6 in LB with 200 mg/ml sucrose at 37 °C with adding the nutrient (LB + 20%sucrose) and removing the culture at 24, 48 and 72 h. **A** sugar concentration and levan in the supernatant of culture; **B** total amount of levan pellets (detected at retention time of 13 and 15 min)

For the issue of glucose inhibition, the continuous cultivation was compared with the batch and fed-batch cultivations. Sucrose was added into the culture at 24, 48 and 72 h and was used completely within 24 h after adding sucrose. In parallel, the glucose concentration was reached over 200 mg/mL at 48 h (Fig. 3). Approximately 60 mg/mL of the glucose was removed from the culture, maintaining a glucose concentration of about 150 mg/mL in the cultivation. At this point, levan production began and continued for 4 days; the results indicated that the levansucrase was either not inhibited at higher glucose concentration or more tolerant of these conditions. Levan yield increased dramatically at 4 days of cultivation with high productivity (Table 1). The continuous system proved the best condition for controlling growth, substrate consumption, enzyme activity and levan production.

### Characterization of purified levansucrase from *B. velezensis* KKSB6

**Fig. 4:**
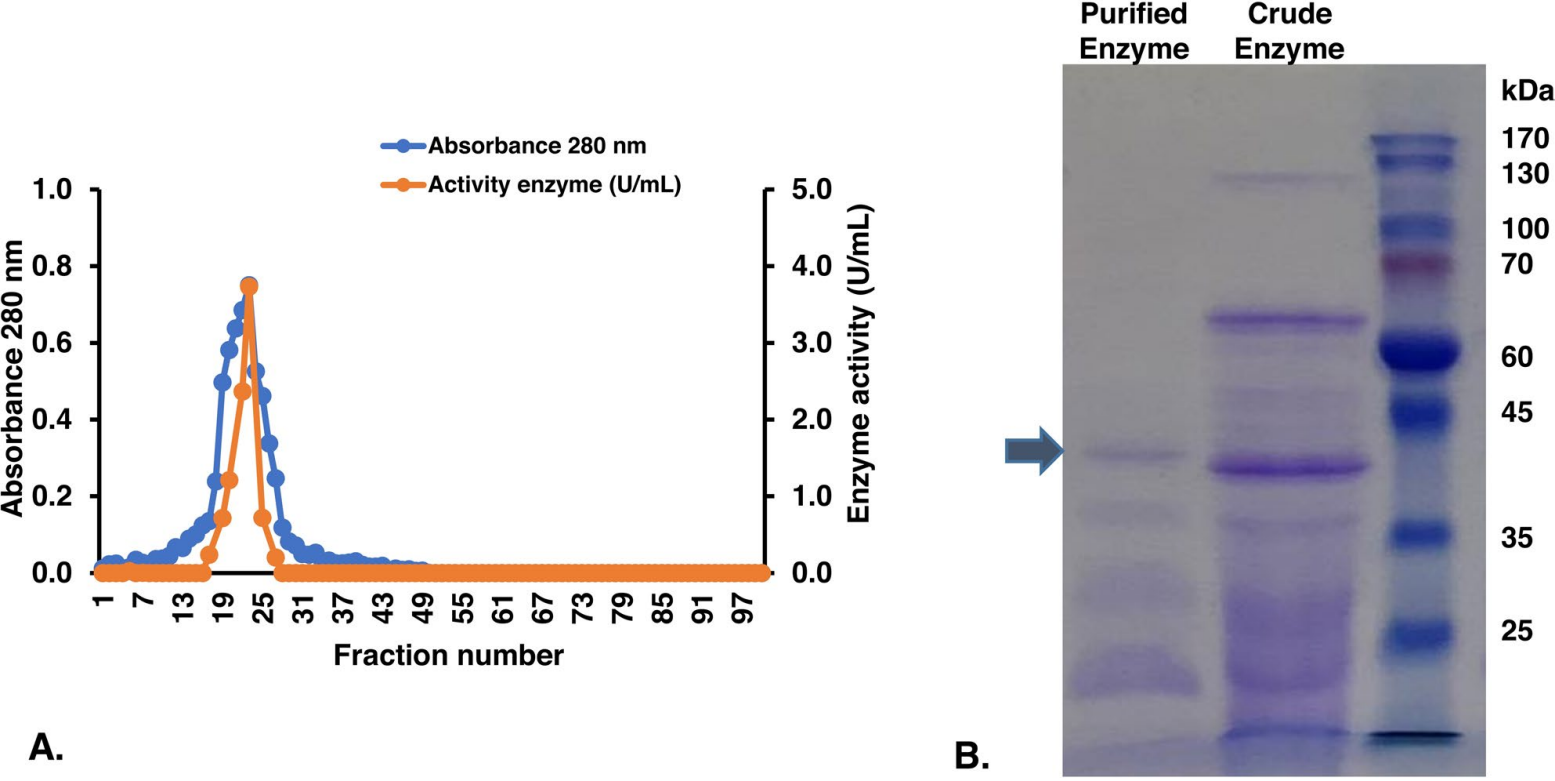
Levansucrase purification using an ion exchange chromatography. **A** fractions represent protein and enzyme activity; **B** protein profiles of crude enzyme and purified enzyme on SDS-PAGE with protein marker from PiNK Plus Prestained Protein Ladder (GeneDireX)

To characterize the enzyme responsible for levan production, levansucrase was concentrated from the culture supernatant and purified using ion exchange chromatography. Fractions 17–24, which exhibited the highest activity, were pooled (Fig. 4A). This purification step resulted in a 7.77-fold purification with a 29.96% yield (Table 2). Analysis of the partially purified enzyme by SDS-PAGE revealed a prominent band with an estimated molecular weight of 42 kDa (Fig. 4B). This partial purified enzyme demonstrated better levan production than the crude enzyme which obtained glucose inhibiting enzyme activity. Thus, this enzyme was used in further steps for levan production.

**Table 2:** Summary of enzyme purification

	Activity (U/ml)	Protein (mg/L)	Specific activity (U/mg)	Yield (%)	Purification (fold)
Crude enzyme	1.40 ± 0.36	90.90 ± 1.03	15.40	100	1.00
Concentrated enzyme	6.93 ± 0.50	175.73 ± 2.61	39.44	193	2.56
DEAE column	3.26 ± 0.44	27.23 ± 0.46	119.72	29.96	7.77

**Fig. 5:**
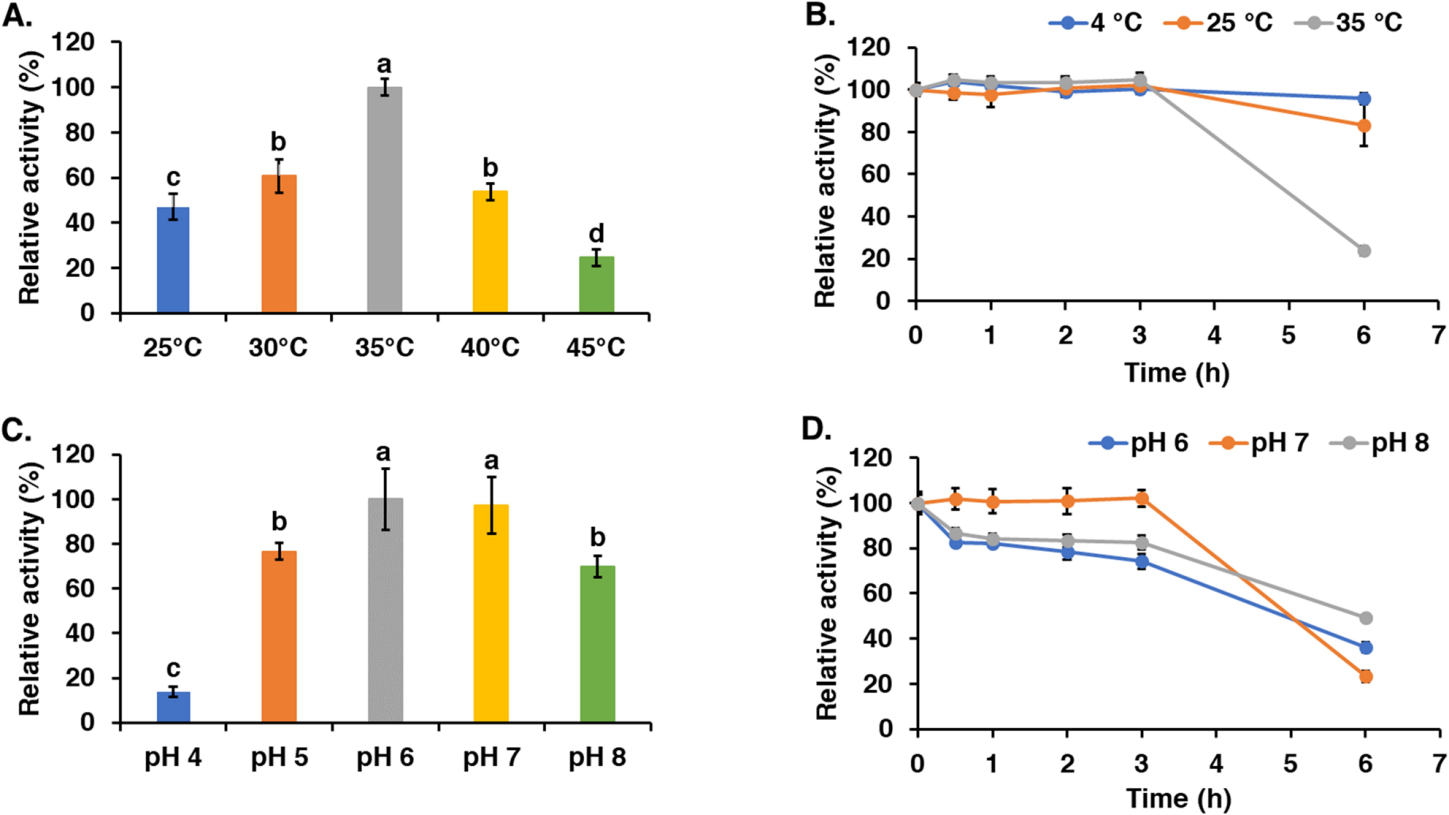
Property of the partial purified levansucrase: **A** optimal temperature; **B** temperature stability; **C** optimal pH; **D** pH stability. The enzyme activity was determined at different temperatures and pHs at 10 min for the optimal conditions. The stability was performed by incubating the enzyme at different temperature and pH in various time to determine the remaining enzyme activity. The experiments were done in triplicate shown in error bars. Different letters on each bar indicate significant differences at *p* < 0.05

**Fig. 6:**
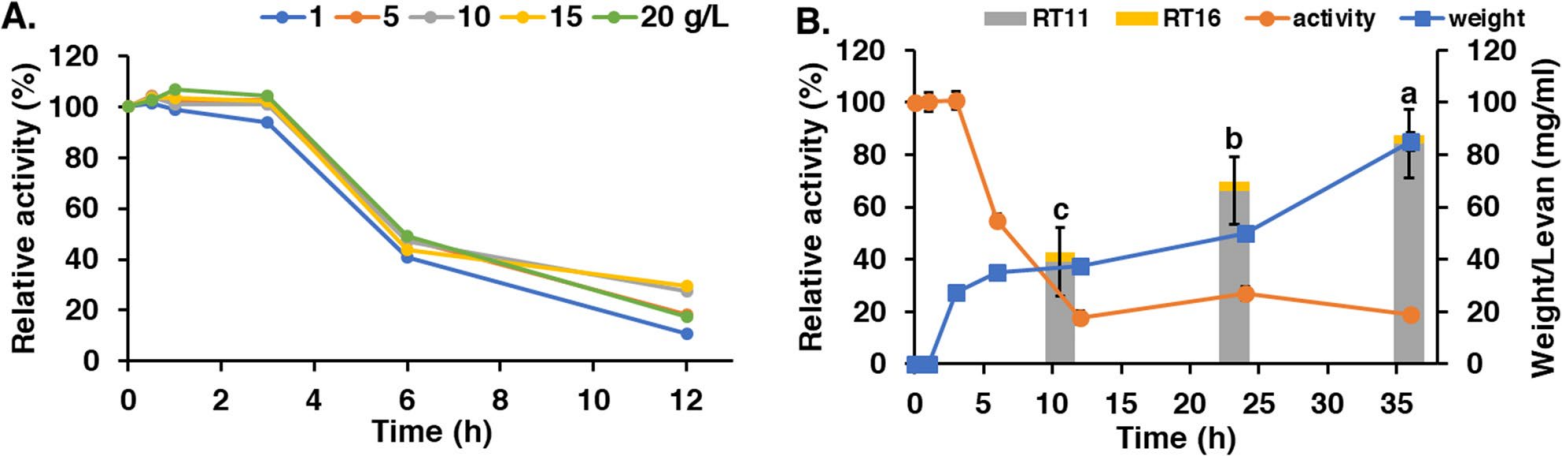
Levansucrase activity and levan production from the purified enzyme. (A) different sucrose concentration (g/L); (B) enzyme activity, levan weight and levan content from the purified enzyme by HPLC at retention time 11 and 16 min (RT11 and RT16). The experiments were done in triplicate shown in error bars. Different letters on each bar indicate significant differences at *p* < 0.05

In order to investigate the optimal enzyme condition for high levan product, the purified levansucrase were analyzed according to the parameters involved in levan production: temperature, pH and substrate concentration. The enzyme exhibited an optimal temperature of 35 °C and an optimal pH in the range of 6.0 to 7.0 (Fig. 5). The enzyme was stable at 35 °C for 3 h before its activity began to decline sharply. In contrast, it showed greater stability at 4 °C and 25 °C, retaining most of its activity for over 6 h. The enzyme was most stable at pH 7.0, retaining activity for up to 3 h. Substrate concentration was also evaluated as a key criterion for levan production. *B. velezensis* KKSB6 levansucrase activity was not inhibited by sucrose concentration up to 20 g/L during 3 h reaction (Fig. 6A). However, after this period, activity decreased, with only 20% residual activity remaining after 12 h, which is consistent with the enzyme’s stability profile. These results imply that levansucrase can function effectively at high sucrose concentrations for short durations.

### Levan production using the purified levansucrase from *B. velezensis* KKSB6

The capacity of the purified levansucrase to produce levan in vitro was tested in a reaction with 20 g/L sucrose. The enzyme activity profile was consistent with the characterization data, decreasing significantly after the 3 h mark (Fig. 6B). Despite the decline in enzyme activity, levan concentration continued to increase over the 12 h reaction period. HPLC analysis of the reaction product revealed two distinct molecular mass fractions, with peaks recorded at retention times of 11 and 16 min (Fig. 6B). These peaks correspond to high-molecular-weight (HMW) levan and low-molecular-weight (LMW) products (levan or fructo-oligosaccharides), respectively. This result suggests that the purified enzyme synthesizes a mixture of levan polymers with varying degrees of polymerization (DP). The in vitro reaction achieved a levan productivity of 2.44 mg/mL/h with a 43.84% yield from the initial sucrose (Table 1).

## Discussion

*Bacillus velezensis* KKSB6, previously isolated from fermented soybeans (Thua nao), has been shown to produce a high-molecular-weight levan (~ 10^4^–10^5^ kDa) with antioxidant and prebiotic properties (Wannasutta et al. 2025). Building on this previous work, the current study aimed to optimize levan production via levansucrase, which is an extracellular enzyme. The results showed that batch cultivation was an effective strategy for achieving a high final yield (187 g/L), while a fed-batch strategy with a single sucrose addition at 24 h resulted in the highest productivity (3.46 g/L/h), albeit with a lower final yield (166 g/L). This reduction can be explained by the dynamics of substrate utilization and product inhibition. In the rapid fed-batch process, sucrose was quickly converted to levan within 48 h, but further production was hindered by the accumulation of glucose, which is known to inhibit both the hydrolysis and transfructosylation activities of levansucrase (Phengnoi et al. 2022). In contrast, the slower sucrose hydrolysis in the batch culture allowed for a longer duration of effective levansucrase activity, resulting in a higher final levan yield before glucose reached inhibitory concentrations. Notably in this study, *B. velezensis* KKSB6 achieved a levan yield of over 50% sucrose conversion, resulting in higher levan production compared to other native strains reported in literature, such as *Bacillus subtilis* AF17 (7.9 g/L) (Bouallegue et al. 2020), *Acetobacter xylinum* NCIM2526 (13.25 g/L) (Srikanth et al. 2015), *Pseudomonas mandelii* (36.7 g/L) (Koşarsoy Ağçeli and Cihangir 2020), *Paenibacillus* sp. strain FP01 (89.5 g/L) (Cheng et al. 2021), and *Pantoea agglomerans* ZMR7 (28.4 g/L) (Al-Qaysi et al. 2021). Furthermore, the 187 g/L yield from batch cultivation of *B. velezensis* KKSB6 was even higher than that achieved by an engineered recombinant *Bacillus amyloliquefaciens* NKΔLP-Y (modified gene for high enzyme expression and secretion), which produced 51 g/L in batch and 102 g/L in fed-batch cultivations (Gu et al. 2017). The glucose inhibition issue could be solved by continuous cultivation processes that maintain low glucose concentration; in fact glucose concentration remained high with higher sucrose addition during continuous cultivation. However, it is likely that over 100 mg/L of glucose did not inhibit levansucrase in continuous cultivation as the levan yield was increased to over 3.5 fold of the fed-batch cultivation with high productivity. Evidently, the molecular mechanism of glucose inhibition should be explored. The continuous strategy employed maintained nutrient for growth and product at the suitable dilution rate and reduced by-product inhibition; thus, this system should be further studied for optimization. Alternatively, genetic engineering approaches could be used to overcome glucose inhibition. For instance, co-expression of a levanase (from *Bacillus licheniformis*) with levansucrase (*Bacillus subtilis*) has been shown to mitigate glucose accumulation (Ávila-Fernández et al. 2023), while engineering host strains to co-produce a value-added product from glucose, such as single-cell oil (*Yarrowia lipolytica*), could also be a viable strategy (Tian et al. 2024).

**Fig. 7:**
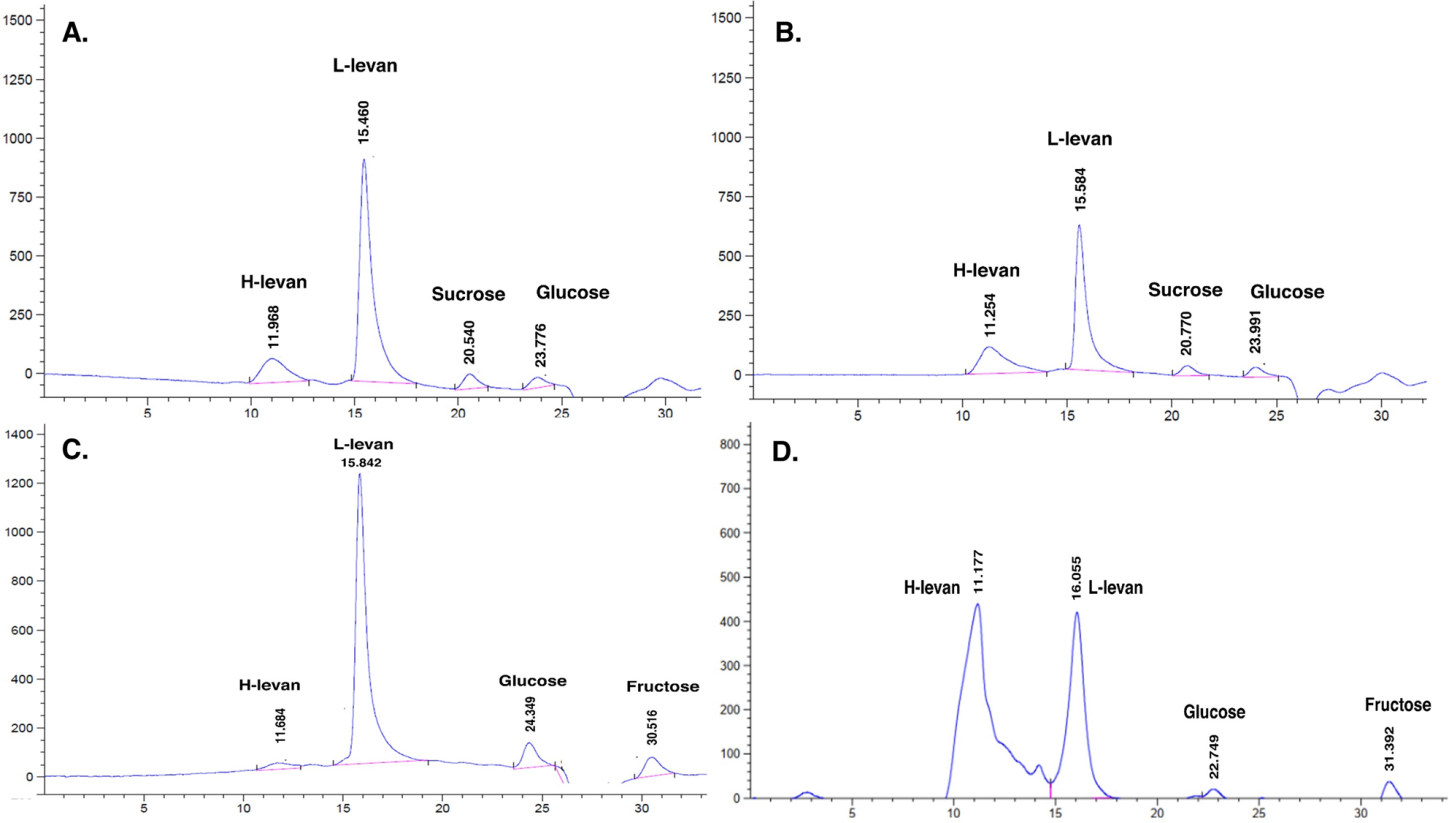
HPLC chromatogram of levan production in different conditions. **A** batch cultivation; **B** fed-batch cultivation; **C** continuous cultivation; **D** enzymatic production at 36 h. H-levan means high molecular weight levan; L-levan means low molecular weight levan

A key finding of this study was the difference in the molecular weight of the levan produced. In vitro synthesis using the purified enzyme primarily yielded high-molecular-weight levan, as indicated by a major peak at a retention time of 10–11 min in HPLC analysis. In contrast, levan produced during whole-cell cultivation appeared to be a lower molecular weight, illustration at 13–16 min of retention time (Fig. 7). This observation suggests that during cultivation, the levan produced may be concurrently hydrolyzed into shorter-chain polymers. This could be due to other secreted enzymes, such as a levanase (encoded by the *yveB* gene often found with *sacB*). This is consistent with previous reports where levan hydrolysis leads to a shift in retention time towards that of lower molecular weight oligosaccharides (Shimizu et al. 2020). Another reason is the changes in culture conditions over time; it may be difficult to continue making long chain polysaccharides so a new strand of oligosaccharide could instead be started, particularly in fed-batch and continuous cultivations. Interestingly, our result contrasts with a study on an immobilized levansucrase from *Zymomonas mobilis*, which produced primarily low-molecular-weight levan (Jang et al. 2001). This difference likely stems from the distinct properties of the enzymes and the reaction conditions used. Given that the *B. velezensis* KKSB6 levansucrase demonstrated good stability for 3 h, enzyme immobilization presents an interesting avenue for developing a robust, recyclable biocatalyst for controlled levan synthesis.

Using the purified enzyme for in vitro synthesis offers the advantage of decoupling levan production from the complexities of cellular metabolism. This allows for the optimization of reaction parameters, such as substrate and enzyme concentrations, without the limitations imposed by cell growth and inhibition. The optimal conditions for the purified enzyme (35–37 °C, pH 6.0–7.0) align with conditions known to favor transfructosylation over hydrolysis (Phengnoi et al. 2022). This finding is supported by the low accumulation of free fructose in our reactions, suggesting that fructose released from sucrose hydrolysis was efficiently transferred to the growing levan chain. The dramatic decrease in enzyme activity observed after the initial reaction period can be attributed to product inhibition by glucose and fructose. However, it is possible that even with reduced hydrolytic activity, the transfructosylation reaction continued, elongating existing levan chains; this would explain the sustained increase in HMW levan.

The molecular weights of levansucrases from *Bacillus* species have been reported to range from 14 kDa to 60 kDa (Phengnoi et al. 2022). The levansucrase from *B. velezensis* BM-2, for example, has a reported molecular weight of approximately 53.5 kDa and exhibits optimal activity at 50 °C and pH 5.6 (Xu et al. 2021), differing from the enzyme in our study. The 42 kDa molecular weight of the *B. velezensis* KKSB6 levansucrase is, however, similar to that of the levansucrase from *Bacillus licheniformis* MJ8, which was reported to be 43 kDa (Omar and Awda 2023). Despite these variations in size and optimal conditions, levansucrases share highly conserved amino acid residues responsible for substrate binding and the catalytic transfer of fructose, placing the enzyme from *B. velezensis* KKSB6 firmly within this important class of fructosyltransferases (Xu et al. 2021; Bakar and Kaplan 2017).

## Conclusion

*Bacillus velezensis* KKSB6 is an exceptional native producer of levan, which was able to create levansucrase for levan production. Continuous cultivation was identified as the optimal method for achieving the highest levan yield (746 g/L) and productivity (7.77 g/L/h). The accumulation of glucose inhibited levansucrase activity and limited overall levan synthesis. The levansucrase from this strain, with a molecular weight of 42 kDa, was characterized and found to exhibit optimal activity at 35 °C and neutral pH. Significantly, in vitro synthesis using the purified enzyme produced primarily high-molecular-weight levan, in contrast to the lower-molecular-weight product observed during fermentation. However, the levan yield from in vitro synthesis was lower than that from continuous cultivation. Therefore, the optimal strategy for levan production from *B. velezensis* KKSB6 depends on the molecular weight of levan desired.

## Supplementary Information

Below is the link to the electronic supplementary material.

Supplementary Material 1

## Data Availability

Data is available in Supplementary Information.
