# Supplementary material for: Analysis of cultivation conditions and enzymatic efficiency enables high-yield levan production from Bacillus velezensis KKSB6
Source: Bioresour Bioprocess. 2025 Dec 26;12(1):153. doi: 10.1186/s40643-025-00994-2 (PMC12741017; doi:10.1186/s40643-025-00994-2)
Supplement: Supplementary file 1 — Supplementary Material 1 [file 40643_2025_994_MOESM1_ESM.docx]

**Supplementary data**

Figure S1 Growth (open circles) and levansucrase activity (close circles) during fed-batch cultivation of *Bacillus velezensis* KKSB6 in LB with 200 mg/ml sucrose at 37^o^C that was added the nutrient (LB+20%sucrose) at 24 h (T1), 48 h (T2), 72 h (T3) and 3 times at 24, 48, 72 h (T4).


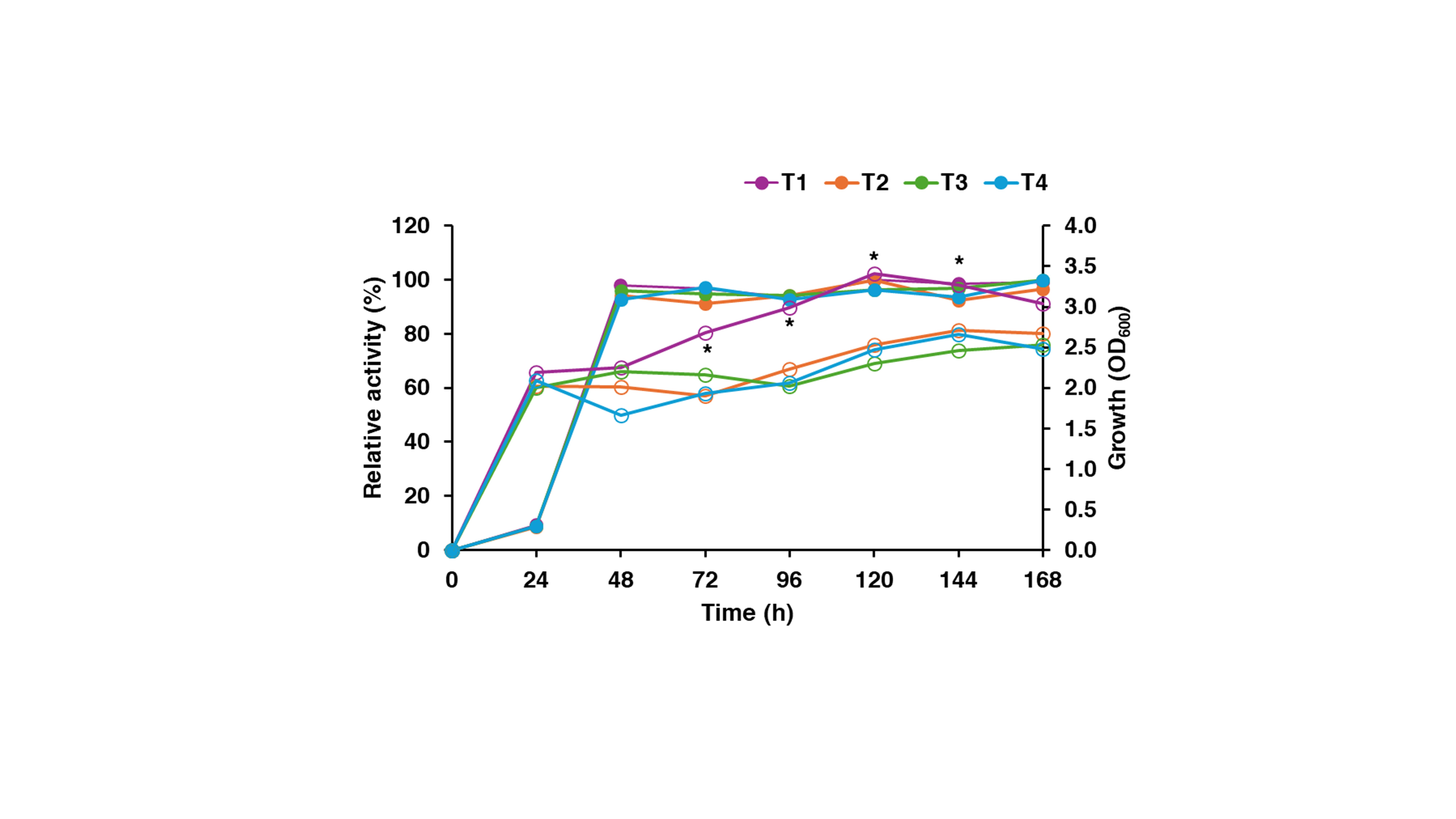


Figure S2 Original photo of protein profiles on SDS-PAGE. Lane 1: crude enzyme; 2 and 5: purified enzyme; 3 and 6: crude enzyme filtered through 3kDa of molecular weight cut-off (Millipore); 4: protein marker from PiNK Plus Prestained Protein Ladder (GeneDireX).


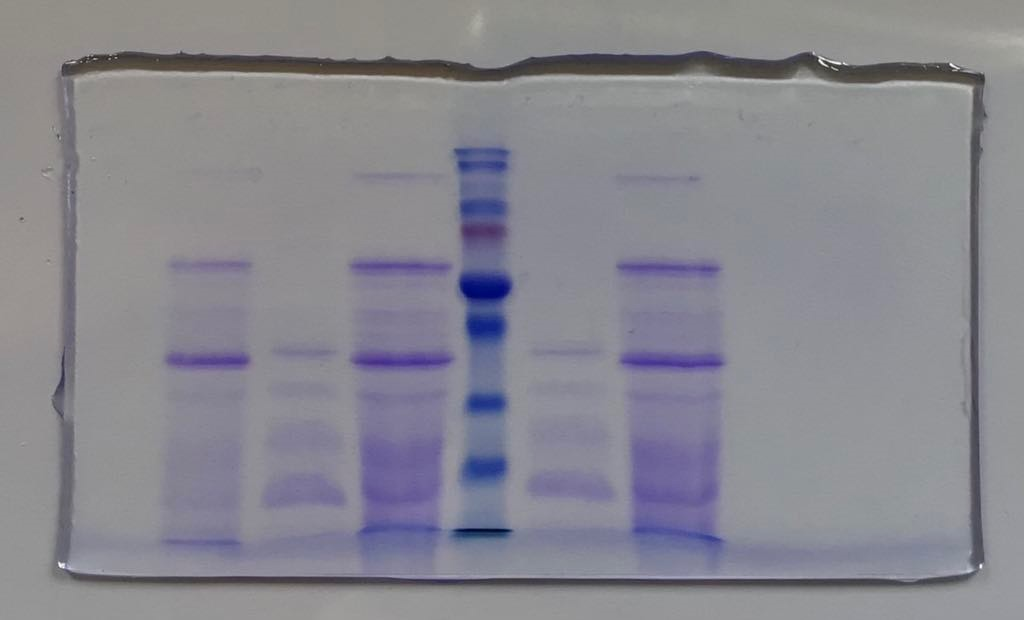


**Lane 1 2 3 4 5 6**
